# Mucin glycan foraging in the human gut microbiome

**DOI:** 10.3389/fgene.2015.00081

**Published:** 2015-03-19

**Authors:** Louise E. Tailford, Emmanuelle H. Crost, Devon Kavanaugh, Nathalie Juge

**Affiliations:** The Gut Health and Food Safety Institute Strategic Programme, Institute of Food ResearchNorwich, UK

**Keywords:** gastrointestinal tract, gut bacteria, intestinal mucus, mucin degradation, *O*-glycosylation, glycoside hydrolase, carbohydrate, gut health and disease

## Abstract

The availability of host and dietary carbohydrates in the gastrointestinal (GI) tract plays a key role in shaping the structure-function of the microbiota. In particular, some gut bacteria have the ability to forage on glycans provided by the mucus layer covering the GI tract. The *O*-glycan structures present in mucin are diverse and complex, consisting predominantly of core 1-4 mucin-type *O*-glycans containing α- and β- linked N-acetyl-galactosamine, galactose and N-acetyl-glucosamine. These core structures are further elongated and frequently modified by fucose and sialic acid sugar residues via α1,2/3/4 and α2,3/6 linkages, respectively. The ability to metabolize these mucin *O*-linked oligosaccharides is likely to be a key factor in determining which bacterial species colonize the mucosal surface. Due to their proximity to the immune system, mucin-degrading bacteria are in a prime location to influence the host response. However, despite the growing number of bacterial genome sequences available from mucin degraders, our knowledge on the structural requirements for mucin degradation by gut bacteria remains fragmented. This is largely due to the limited number of functionally characterized enzymes and the lack of studies correlating the specificity of these enzymes with the ability of the strain to degrade and utilize mucin and mucin glycans. This review focuses on recent findings unraveling the molecular strategies used by mucin-degrading bacteria to utilize host glycans, adapt to the mucosal environment, and influence human health.

## Introduction

The human gastrointestinal (GI) tract harbors a complex and dynamic population of microorganisms which contribute significantly to the maintenance of health and the onset and progression of disease (Sommer and Backhed, 2013). The intestinal epithelium surface is covered by a layer of mucus which differs in terms of composition, organization, and thickness along the GI tract (Pullan et al., 1994; Linden et al., 2008; Johansson et al., 2011; Juge, 2012; Ermund et al., 2013). In the colon, the mucus is divided into an outer layer which provides a nutrient-rich habitat for the microbiota and an inner layer firmly attached to the surface of the epithelium, and virtually free of bacteria (Johansson et al., 2008, 2011). There is an emerging paradigm that mucus is critical to the maintenance of a homeostatic relationship between the gut microbiota and their hosts, with subtle deviations from this dynamic interaction potentially resulting in major implications for health, among which are colitis, colorectal cancer, and susceptibility to infection, as extensively reviewed (McGuckin et al., 2011; Hansson, 2012; Sheng et al., 2012; Chen et al., 2014). Recent findings showed that alterations in mucosal carbohydrate availability impact on the composition of microbial species (Martens et al., 2008; Ng et al., 2013; Tong et al., 2014). The mucosal subpopulation is in a prime position to influence host immune responses. This review is focussed on the nutritional strategies used by gut bacteria to proliferate into the mucosal environment.

## Mucins and mucin glycans of the GI tract

### Intestinal mucins

**Figure 1:**
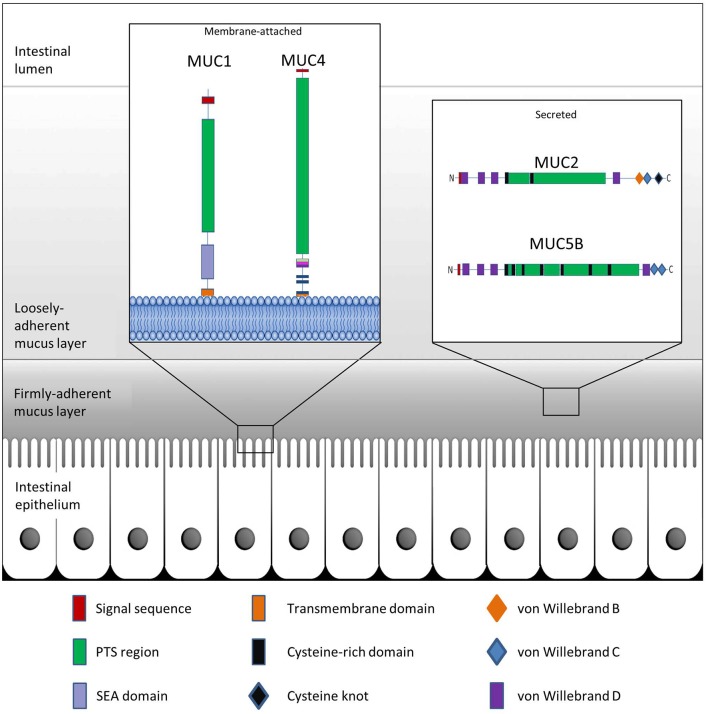
**Schematic representation of GI mucus and mucins**. The colonic epithelium is protected by mucin glycoproteins which are either membrane-attached (e.g., MUC1 and MUC4) or are secreted from goblet cells into the intestinal lumen (e.g., MUC2 and MUC5B). The secreted mucins further create a protective boundary in the form of a tightly-adherent mucus layer, which is devoid of bacteria, and a loosely-adherent mucus layer which provides a niche for intestinal bacteria.

Mucins are the main structural components of mucus and play an integral and multifaceted role in the interaction between microbes and epithelial surfaces (Linden et al., 2008; Johansson et al., 2011). The expression profile of mucins varies among host tissues and particularly within the GI tract, which displays the highest and most diverse levels of mucin expression in the body (Linden et al., 2008). To date, more than 20 genes encoding mucins have been identified in humans, with their classification based on the arrangement of their monomeric polypeptide domains (Corfield, 2015). Mucins are broadly grouped as membrane-bound or secreted proteins (Moran et al., 2011; Corfield, 2015) (Table 1). Common to each mucin are an N-terminal signal peptide and a proline-threonine-serine (PTS) domain (Figure 1). The signal peptide is required for the targeting of the polypeptide to the endoplasmic reticulum (ER) and either extracellular secretion or insertion of the synthesized mucin into the cell membrane. The PTS domain is the site of extensive *O*-glycosylation with carbohydrates accounting for up to 80% of the total mucin mass (Gendler and Spicer, 1995). These PTS domains, comprised of variable number of tandem repeat (VNTR) domains, allow for a great degree of heterogeneity in mucins, due to variability in both, mucin length and extent of glycan attachment at these sites. This characteristic filamentous protein backbone decorated with outwardly protruding oligosaccharides results in the typical “bottle-brush”-like appearance of mucins (Bergstrom and Xia, 2013).

**Table 1:** **Human membrane-bound and secreted GI mucins**.

**Type**	**MUC Gene**	**Tissue localization (Detection method)**	**References**
Membrane	MUC1	Stomach^a^ Duodenum, colon^a^,^b^	Ho et al., 1993; Buisine et al., 2000a,b
	MUC3A/B	Goblet and absorptive cells^b^; Jejunum, ileum and colon^a^; small intestinal columnar cells and surface colonic epithelium^b^	Audie et al., 1993; Ho et al., 1993; Chang et al., 1994
	MUC4	Stomach and colon^a^	Porchet et al., 1995
	MUC12	Stomach, small intestine, and colon^a^	Packer et al., 2004
	MUC13	Small intestine and colon^a^	Packer et al., 2004
	MUC15	Small intestine and colon^a^	Pallesen et al., 2002
	MUC17	Stomach, small intestine (highest expression in duodenum) and colon (transverse)^a^	Gum et al., 2002
	MUC20	Colon^a^	Higuchi et al., 2004
	MUC21	Colon^a^	Itoh et al., 2008
Secreted	MUC2	Small intestine (jejunum and ileum) and colon^a^; Goblet cells of small intestine and colon^b^	Audie et al., 1993; Ho et al., 1993
	MUC5AC	Stomach^a^	Porchet et al., 1995; Buisine et al., 2000b
	MUC5B	Colon^a^ (weakly expressed)	Porchet et al., 1995
	MUC6	Stomach^a^ (glands)^b^, duodenum (Brunner glands)^b^	Buisine et al., 2000b; Reis et al., 2000; Nordman et al., 2002

a *Detection by gene expression (mRNA expression, Northern blot, or in situ hybridization)*.

b *Detection by immunohistochemistry*.

Membrane-bound mucins are essential contributors of the glycocalyx of mucosal surfaces where they play important biological roles in cell-cell and cell-matrix interactions, and in cell signaling (Jonckheere et al., 2010). These mucins may be shed from the surface and integrate into the overlying mucus layer where they are able to influence the viscosity of the protective layer (Carrington et al., 2009). Secreted mucins are the main structural components of the mucus gel. Along the GI tract, synthesis and secretion of these polymeric glycoproteins take place in the goblet cells of the small intestine and colon, or the surface mucous cells of the stomach (Moncada et al., 2003). Characteristic properties of the secreted mucins are disulfide bridges which are formed among the cysteine-rich, cysteine knot, and von Willebrand C and D domains residing at the N- or C-termini of the glycoprotein monomers (Desseyn et al., 2008; Ambort et al., 2011) (Figure 1). MUC2 is the best characterized secreted mucin of the GI tract (Nilsson et al., 2014). Within the ER, newly synthesized MUC2 peptides immediately dimerize through the formation of disulfide bridges followed by transport to the Golgi apparatus (Asker et al., 1995, 1998; Ambort et al., 2012b). Here, the PTS domains of the mucin dimers are sites of elaborate glycosylation before further assembly into trimers in the trans-Golgi network and packaging into goblet cell vesicles in a pH- and Ca^2+^-dependent manner (Godl et al., 2002; Ambort et al., 2012a; Johansson and Hansson, 2012). As a monomer, fully glycosylated MUC2 exhibits a large size of approximately 2.5 MDa, while extensive polymerization may allow for sizes of greater than 100 MDa (Johansson et al., 2011). Following secretion of the mucin granules at the mucosal surface, the densely-packed mucin structures are hydrated and rapidly expand to a size approximately 3000-fold greater than in the granules, thus providing a dynamic barrier (Verdugo, 2012).

In addition to their protective and lubricating activities, mucins facilitate microbial tropism through the presentation of glycans which may impact colonization (e.g., Kobayashi et al., 2009; Gonzalez-Rodriguez et al., 2012; Etzold et al., 2014, for a review see Juge, 2012), and act as a nutritional source for microorganisms (e.g., Ruas-Madiedo et al., 2008; Crost et al., 2013, for a review see Marcobal et al., 2013). As such, mucin glycans have been proposed to play a key role in selecting microbial communities along and across the GI tract. Consistent with this hypothesis, recent studies in mouse models and humans showed an association between alteration in mucin glycosylation profile and deviations of overall community ecology along with altered abundances of specific microbes (Rausch et al., 2011; Wacklin et al., 2011, 2014; Pacheco et al., 2012; Kashyap et al., 2013; Sommer et al., 2014).

### Mucin glycosylation

**Figure 2:**
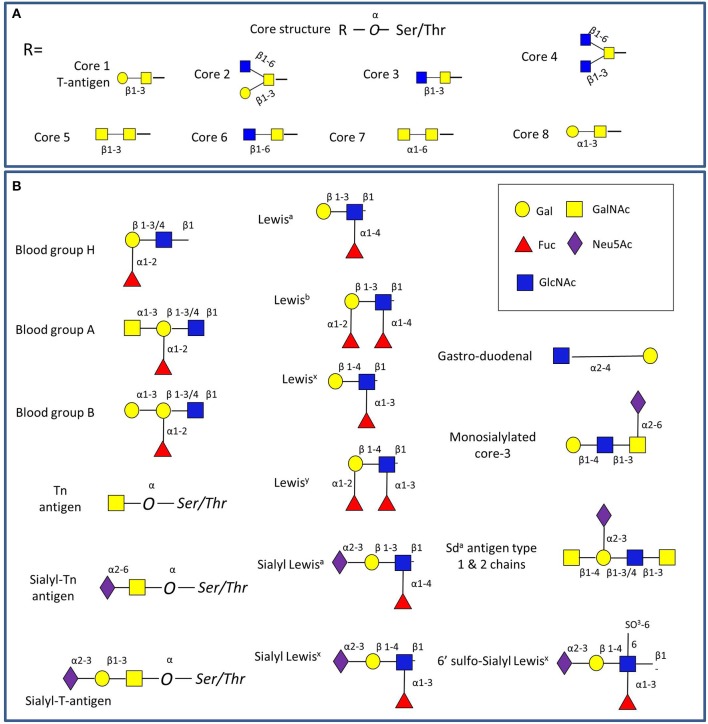
**Schematic representation of GI mucin glycans**. **(A)** The four common mucin type *O*-glycans (core 1-8) found in the GI tract. **(B)** Main glycan epitopes in GI mucins. The glycan sugars are represented using Glycan Builder (Ceronietal., 2007).

Mucins carry a vast array of oligosaccharide structures, with the glycosyltransferase profile expressed by the host determining the type of linkages and glycan structures present on the secreted mucins (Linden et al., 2004). The synthesis of mucin oligosaccharides starts with the transfer of N-acetylgalactosamine (GalNAc) to Ser and Thr residues of the mucin core (Bennett et al., 2012). Eight core structures of the mucin *O*-glycan chain have been identified (Brockhausen et al., 2009), with core 1-4 glycans most commonly found in intestinal mucins (Figure 2A). In humans, gastric and duodenal mucins generally contain the core 1 (Galβ1–3GalNAcα1-Ser/Thr) and the core 2 (Galβ1,3(GlcNAcβ1,6)GalNAcα1-Ser/Thr) structures (Robbe et al., 2004; Larsson et al., 2009), while the core 3 (GlcNAcβ1,3GalNAcαSer/Thr) structure is predominant in the small intestine (Robbe et al., 2004), and core 3 and 4 (GlcNAcβ1,6(GlcNAcβ1,3)GalNAcαSer/Thr) structures make up the majority of colonic mucin glycans (Robbe et al., 2004; Robbe-Masselot et al., 2009; Xia, 2010). Further studies revealed that MUC2 in the sigmoid colon mainly contains the core 3 structure (Robbe et al., 2004; Thomsson et al., 2012). These core structures can be further extended with galactose (Gal), N-acetylglucosamine (GlcNAc), GalNAc, fucose or sialic acid (Neu5Ac) sugar residues with the latter two frequently occupying terminal positions (Brockhausen et al., 2009). Alterations in mucin glycosylation have been associated with a number of diseases such as colitis, colonic cancer and inflammatory bowel diseases in humans (e.g., McGovern et al., 2010; Larsson et al., 2011; Rausch et al., 2011; Parmar et al., 2012; Forni et al., 2014) and mouse models (e.g., An et al., 2007; Stone et al., 2009; Fu et al., 2011). However, more work is needed to support the causal link between altered *O*-linked glycosylation and inflammation, as recently reviewed in Theodoratou and colleagues (Theodoratou et al., 2014).

The main source of glycan diversity is provided by the peripheral terminal epitopes that show considerable variation (Figure 2B). The H1 structure (α1,2-fucose) is found in populations carrying the secretor gene (Mollicone et al., 1985), and individuals may also express the Lewis gene and the Le^b^ histo-blood group antigen if they are secretors, while non-secretors express Le^a^ (Kelly et al., 1995). Another phenotype (SeW—weak secretor) is characterized by the expression of both Le^a^ and Le^b^ antigens (Henry et al., 1995; Lindén et al., 2008). The presentation of the major mucin glycan epitopes, sialic acid and fucose, varies along the GI tract following opposing gradients with a decreasing gradient of fucose and ABH blood group expression and an increasing gradient of sialic acid from the ileum to the colon (Robbe et al., 2004). Interestingly, these gradients are reversed in mice, where the small intestine is dominated by sialylated structures and the colon with those terminating in fucose, potentially creating the need for additional considerations in the comparison of human and murine colonization studies (Holmen Larsson et al., 2013). These terminal mucin *O*-glycans have been proposed to serve as metabolic substrates, providing a nutritional advantage to bacteria which have adapted to the GI mucosal environment (Freitas et al., 2003; Severi et al., 2007; Pacheco et al., 2012; Vimr, 2013). Conversely, gut bacteria have the ability to affect the mucus barrier (Jakobsson et al., 2015) and mucin glycosylation (Hooper et al., 1999; Pickard et al., 2014).

## Mucin-degrading bacteria of the human gut microbiota

The GI tract is heavily colonized by bacteria with most species belonging to the phyla Firmicutes, Bacteroidetes, Actinobacteria, Proteobacteria, and Verrucomicrobia. The microbiota composition varies longitudinally along the GI tract but also transversally from the mucosa to the lumen (Zoetendal et al., 2002; Eckburg et al., 2005; Carroll et al., 2010). Defining the mucosa-associated bacteria composition is hampered by difficulties in (i) sampling, limiting the number and power of human studies, (ii) differences in the nature or definition of the mucosa samples (biopsies, mucus, or rectal swabs), and (iii) intrinsic inter-individual variability at the family, genera, or species level (see for example Hong et al., 2011). However, it is now clear that the composition of the mucosa-associated microbiota differs from that of the fecal microbiota in terms of relative abundance of the different phyla, although the results may vary between studies (Swidsinski et al., 2005; Frank et al., 2007; Carroll et al., 2010). For example, the percentage of Bacteroidetes phylum was shown to be higher in the colonic biopsies or rectal swabs of healthy human volunteers compared to the feces (Eckburg et al., 2005; Chen et al., 2012). In contrast, Firmicutes were enriched in the mucosa-associated bacteria of mice, especially members of the Lachnospiraceae and Ruminococcaceae families (Nava et al., 2011). Similarly, Van den Abbeele and collaborators showed that the Firmicutes phylum, especially members of *Clostridium* cluster XIVa, was significantly enriched in the mucus layer as opposed to the lumen, using an *in vitro* gut model inoculated with human fecal samples (Van Den Abbeele et al., 2013). These differences may be due to the different models used in these studies (e.g., *in vivo* vs. *in vitro*; human vs. mice) and sampling methods. In addition, a number of studies have focused on specific bacterial groups or species. For example, sulfate-reducing bacteria, acetogenic bacteria, and methanogenic archaea were shown to preferentially colonize the healthy human colon mucosa (Nava et al., 2012). An early study reported that *Lactobacillus gasseri* was a predominant *Lactobacillus* species in human biopsy samples (Zoetendal et al., 2002) whereas *Bifidobacterium bifidum* and *Bifidobacterium longum* were shown to be more abundant in the mucosa of germ-free rats inoculated with human fecal microbiota than in the lumen (Van Den Abbeele et al., 2011). *Faecalibacterium prausnitzii*, an abundant member of the microbiota with putative anti-inflammatory properties, has also been found in ileal, colonic, and rectal biopsies from healthy individuals (Lopez-Siles et al., 2014).

In the colon, the epithelium is covered by a thick gel of mucus, divided into two layers, an inner layer firmly attached to the epithelium and a loose outer layer (Atuma et al., 2001; Ermund et al., 2013). Johansson and collaborators demonstrated that the outer mucus layer is heavily colonized by bacteria, while the inner layer contains no or very few bacteria (Johansson et al., 2008). It is thus believed that in healthy conditions mucosa-associated bacteria are not in direct contact with the epithelium but are restricted to the outer mucus layer. Although the molecular mechanisms underpinning the adaptation of gut bacteria to mucus remain unclear, it is likely that their ability to utilize mucin glycans as a source of nutrients would confer a competitive advantage to those bacteria with the required repertoire of hydrolytic enzymes. The first mucin-degrading (or mucinolytic) bacteria studied were pathogens (e.g., Levy and Aminoff, 1980; Prizont, 1982; Slomiany et al., 1992), and thus for a long period mucin degradation had been associated with pathogenicity. However, it is now clear that mucin degradation is part of a normal turn-over process starting a few months after birth (Norin et al., 1985). To date, only a limited number of bacterial species/strains from the Bacteroidetes, Firmicutes, Actinobacteria, and Verrucomicrobia phyla have been studied for their ability to consume mucins (see below and Table 2).

**Table 2:** **Mucin-degrading bacteria colonizing the human GI tract**.

**Bacterial species**	**Strain**	**Mucin tested**	**Enzymatic activities^a^**	**References**
*B. thetaiotaomicron*	VPI-5482 = ATCC 29148	Purified *O*-glycans from PGM type III	α-L-fucosidase; endo-β-N-acetylglucosaminidase; endo-β-galactosidase; α-mannosidase	Martens et al., 2008
*B. fragilis*	2 strains	BSM	Unknown	Salyers et al., 1977a
	ATCC 23745	PGM; pig colonic mucin	Unknown	Roberton and Stanley, 1982
	ATCC 25285	PGM; pig colonic mucin	Unknown	Roberton and Stanley, 1982
		Purified *O*-glycans from PGM type III	Sialidase	Marcobal et al., 2011
*B. vulgatus*	VIII-271F	pPGM^*^	Unknown	Hoskins et al., 1992
	ATCC 8482	pPGM type II	Unknown	Png et al., 2010
*Bacteroides caccae*	1 strain	BSM	Unknown	Salyers et al., 1977a
*A. muciniphila*	ATCC BAA-835	pPGM type III	α-galactosidase; β-galactosidase; α-L-fucosidase; β-glucosidase; α-mannosidase; α-galactosidase; β-D-fucosidase; α-N-acetylgalactosaminidase; β-N-acetylgalactosaminidase; β-N-acetylglucosaminidase; sulfatase	Derrien et al., 2004; Derrien, 2007
		pPGM type II; human MUC2	Unknown	Png et al., 2010
*R. gnavus*	ATCC 29149	pPGM type II; human MUC2	Unknown	Png et al., 2010
		pPGM type III	α-L-fucosidase; α2,3-sialidase	Crost et al., 2013
	ATCC 35913 = VI-268	pPGM^*^	Blood group B-degrading activity; blood group H-degrading activity; sialidase; β-galactosidase; sialate O-acetylesterase	Hoskins et al., 1985; Corfield et al., 1992
		pPGM type III	Unknown	
*R. torques*	IX-70 = ATCC 35915	pPGM^*^	Blood group A-degrading activity; blood group H-degrading activity; sialidase; β-galactosidase; β-N-acetylgalactosaminidase; β-N-acetylglucosaminidase; sialate O-acetylesterase; glycosulfatase	Hoskins et al., 1985; Corfield et al., 1992; Hoskins et al., 1992
	VIII-239	pPGM^*^	Same as for strain IX-70	Hoskins et al., 1985; Corfield et al., 1992; Hoskins et al., 1992
	ATCC 27756	pPGM type II; human MUC2	Unknown	Png et al., 2010
*B. bifidum*	D119	PGM type III	Unknown	Ruas-Madiedo et al., 2008; Turroni et al., 2010
	L22	PGM type III	endo-α-N-acetylgalactosaminidase; α1,2-L-fucosidase	Ruas-Madiedo et al., 2008; Turroni et al., 2010
	VIII-210 = ATCC 35914	pPGM^*^	Blood group H-degrading activity; sialidase; β-galactosidase; β-N-acetylgalactosaminidase; β-N-acetylglucosaminidase; sialate O-acetylesterase; glycosulfatase	Hoskins et al., 1985; Corfield et al., 1992; Hoskins et al., 1992
		pPGM type II; human MUC2	Unknown	Png et al., 2010
	PLR2010	PGM type III	α1,2-L-fucosidase; α1,3/4-L-fucosidase; exo-α-sialidases; α-L-arabinofuranosidase; α1,3-galactosidase; endo-α-N-acetylgalactosaminidase; β-N-acetylhexosaminidases; β-galactosidase	Turroni et al., 2010
	A8		Unknown	Turroni et al., 2010
	324B		Unknown	Turroni et al., 2010
	156B		Unknown	Turroni et al., 2010
	85B		Unknown	Turroni et al., 2010
	DSM 20456		Unknown	Turroni et al., 2010
*B. longum subsp. longum*	NCIMB8809	PGM type III	Unknown	Ruas-Madiedo et al., 2008
		Human intestinal mucus	β-N-acetylglucosaminidase; β-glucuronidase	Ruiz et al., 2011
*B. longum subsp. infantis*	VIII-240	pPGM^*^	Blood group H-degrading activity; sialidase; β-galactosidase; β-N-acetylgalactosaminidase; β-N-acetylglucosaminidase; sialate O-acetylesterase; glycosulfatase	Hoskins et al., 1985; Corfield et al., 1992; Hoskins et al., 1992
	ATCC 15697	PGM type III	Unknown	Turroni et al., 2010; Kim et al., 2013
*B. breve*	NCIMB8807	PGM type III	Unknown	Ruas-Madiedo et al., 2008

*PGM, pig gastric mucin (Sigma-Aldrich)*.

PGM^*^, pig gastric mucin (ICN Nutritional Biochemicals).

*pPGM, purified PGM, purified according to Miller and Hoskins' method (Miller and Hoskins, 1981)*.

*BSM, bovine submaxillary mucin (Sigma-Aldrich)*.

a *Enzymatic activities putatively involved in mucin degradation were identified by (i) activity assays using the spent media, (ii) activity assays using the lysed cells or (iii) transcriptomic assay, when the bacterium was grown with mucin*.

The mucin-degrading ability of gut bacteria has been extensively studied in Bacteroidetes. An early study showed that all 22 strains of *Bacteroides thetaiotaomicron* tested were able to ferment glycosaminoglycans (GAG) but failed to ferment pig gastric mucin (PGM) or bovine submaxillary mucin (BSM) (Salyers et al., 1977a). However, later, *B. thetaiotaomicron* VPI-5482 was shown to be able to grow on different fractions of glycans purified from pig gastric mucosa, including an *O*-glycan rich fraction (Martens et al., 2008). Transcriptomic analyses highlighted specific polysaccharide-utilization loci (PULs) including genes coding for putative glycoside hydrolases (GHs) such as α-L-fucosidase, endo-β-N-acetylglucosaminidase, endo-β-galactosidase and α-mannosidase, which were up-regulated when *B. thetaiotaomicron* was grown on mucin *O*-glycans or in monoxenic mice as compared to *in vitro* glucose control. Interestingly, these PULs were not up-regulated when *B. thetaiotaomicron* was grown on GAG, as compared to glucose (Martens et al., 2008, 2011). Colonization competition experiments demonstrated that *B. thetaiotaomicron* mutants for *O*-glycan PULs were able to colonize germ-free mice in a similar way as the wild-type strain when mice were fed a plant glycan-rich diet, but were outcompeted by the wild-type on a simple-sugar diet (Martens et al., 2008). This indicates that *B. thetaiotaomicron* relies on mucin and other host-derived glycans for colonization. Genome analysis of *Bacteroides fragilis* confirmed that *Bacteroides* species contain a much larger number of genes encoding carbohydrate-active enzymes (CAZymes) compared to other sequenced gut bacteria (Kuwahara et al., 2004). In accordance with early studies demonstrating the ability of some *B. fragilis* strains to grow on mucin as sole carbon source (Salyers et al., 1977a; Roberton and Stanley, 1982), the *B. fragilis* genome contains a subset of PULs dedicated to host mucin *O*-glycan utilization; in particular, it has been shown that (i) loci involved in the binding, degradation, and transport of sialylated polysaccharides play an important role in the colonization of this bacterium in the gut (Nakayama-Imaohji et al., 2012) and (ii) the genes involved in sialic acid utilization are up-regulated when *B. fragilis* is grown in the presence of mucin *O*-glycans as compared to glucose (Marcobal et al., 2011). Some strains of *Bacteroides vulgatus* have also been shown to moderately degrade PGM but failed to utilize human MUC2 (Hoskins et al., 1992; Png et al., 2010) (Table 2).

In the Firmicutes phylum, *Ruminococcus torques* and *Ruminococcus gnavus*, both members of the Lachnospiraceae family (belonging to the *C. coccoides* group/cluster XIVa) have been shown to degrade mucins. In an early study, six *R. torques* strains out of nine tested, but none of the *R. gnavus* strains tested, were shown to have the capacity to ferment PGM (Salyers et al., 1977b). A few years later, *R*. *gnavus* ATCC 35913 and two *R. torques* strains (ATCC 35915 and VIII-239) were among the five strains isolated from human fecal samples for their ability to degrade mucins (Hoskins et al., 1985). Png and collaborators then confirmed that both *R*. *gnavus* and *R. torques* species were able to degrade and utilize human MUC2 as a sole carbon source (Png et al., 2010), providing further evidence of their adaptation to the human colonic mucosal environment. Several enzymatic activities were detected in the spent media of these strains grown with mucin that could explain their ability to degrade mucin (Hoskins et al., 1985, 1992; Corfield et al., 1992; Crost et al., 2013) (Table 2). Recently, the ability of *R. gnavus* strains to utilize mucin was shown to be strain-dependent, as also supported by comparative genomic and transcriptomic analyses (Crost et al., 2013), and in agreement with earlier findings (Salyers et al., 1977b).

*B. bifidum* ATCC 35914 and *B*. *longum* subsp. *infantis* VIII-240, from the Actinobacteria phylum, were also isolated by Hoskins and collaborators (Hoskins et al., 1985) as mucin degraders, and several enzymatic activities possibly involved in mucin degradation were detected in the spent media of these strains grown with mucin as sole carbon source (Hoskins et al., 1985, 1992; Corfield et al., 1992) (Table 2). Since then, *B*. *longum* subsp. *infantis* has been shown to grow on mucins, albeit moderately (Abe et al., 2010; Turroni et al., 2010; Kim et al., 2013), and the ability of *B. bifidum* to utilize mucins has been confirmed for several strains using different types of mucins, including human MUC2 (Ruas-Madiedo et al., 2008; Png et al., 2010; Turroni et al., 2010) (Table 2). Transcriptomic analyses of *B. bifidum* L22 and PLR2010 suggest the involvement of several enzymes in the process, e.g., α-L-fucosidase and endo-α-N-acetylgalactosaminidase (Ruas-Madiedo et al., 2008; Turroni et al., 2010) (Table 2). Few other Bifidobacteria species, including some strains of *B*. *longum* subsp. *longum* and *Bifidobacterium breve*, have also been shown to degrade mucins *in vit*ro (Ruas-Madiedo et al., 2008; Turroni et al., 2010; Ruiz et al., 2011) (Table 2). β-N-acetylglucosaminidase and β-glucuronidase activities were increased in the spent medium of *B*. *longum* subsp. *longum* NCIMB8809 grown with human intestinal mucus, suggesting a role of these activities in mucin degradation. More recently, detailed genome analyses of Bifidobacteria have identified metabolic pathways for the degradation of mucin-type *O*-glycans and human milk oligosaccharides (HMOs) (Sela et al., 2008; Turroni et al., 2010) and several GHs have been functionally characterized supporting these findings (Yamamoto et al., 2010; Kiyohara et al., 2011) (see section below).

In the Verrucomicrobia phylum, *Akkermansia muciniphila*, a strictly anaerobic Gram-negative bacterial species, was recently identified as a key mucin degrader (Derrien et al., 2004). Initially isolated from a human fecal sample due to its ability to utilize mucins as sole carbon and nitrogen source, *A. muciniphila* has since been shown to be a common member of the human gut with a high prevalence and variable abundance, present both in feces and at the mucosal surface (Eckburg et al., 2005; Collado et al., 2007; Derrien et al., 2008). When *A. muciniphila* ATCC BAA-835 was grown with mucin as sole carbon source, several enzymatic activities potentially involved in mucin degradation were detected both in the spent medium and intracellularly (Derrien, 2007). However, although numerous genes encoding putative mucinolytic enzymes were found in the genome of the ATCC BAA-835 strain (Van Passel et al., 2011), the functional characterization of these proteins, and thus their role in mucin degradation, has not yet been reported.

## Bacterial enzymes involved in mucin degradation

**Figure 3:**
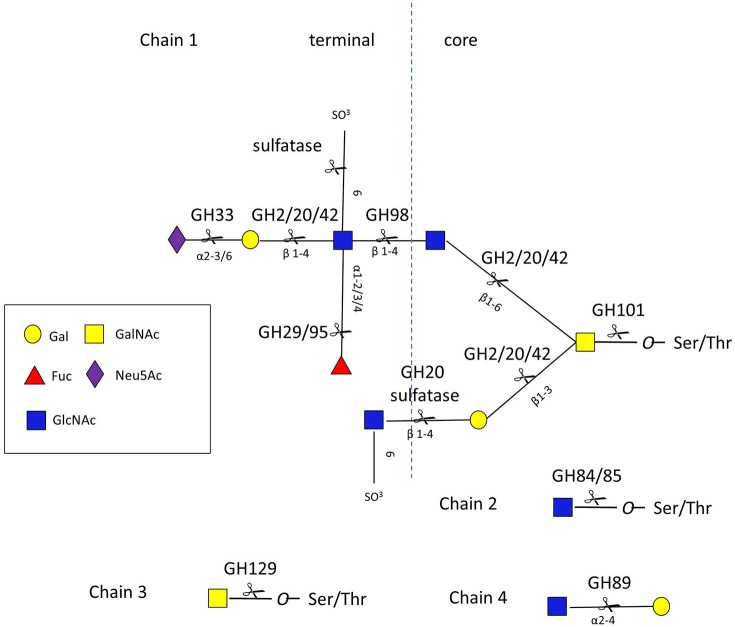
***O*-glycan chains showing sites of action of GHs and sulfatases**. Chain 1 is a hypothetical mucin glycan chain, chain 2 is *O*-GlcNAc often found on other glycoproteins, chain 3 (Tn antigen) and chain 4 are found in gastro-duodenal mucin.

Given the diversity and complexity of intestinal mucin glycan structures, strategies for deconstructing these molecules rely on the cooperative action of a number of proteases, sulfatases, and GHs encoded by the genome of mucin-degrading bacteria. These GHs include, but are not limited to, neuraminidases/sialidases (GH33), fucosidases (GH29 and GH95), exo- and endo-β-N-acetylglucosaminidases (GH84 and GH85), β-galactosidases (GH2, GH20, and GH42), α-N-acetylglucosaminidases (GH89), and α-N-acetylgalactosaminidases (GH101, GH129) (www.cazy.org/) (Lombard et al., 2014) (Figure 3 and Table S1).

However, in sharp contrast to the number of genes predicted to be involved in mucin degradation (mainly based on transcriptomic analyses), only a few GHs have been biochemically characterized (see CAZy database; www.cazy.org). Detailed enzymatic characterization of putative mucin-degrading enzymes, and CAZymes in general, is essential for allowing accurate annotation of homologous gene sequences. Indeed, the process of reliable annotation of protein function from gene sequence is one of the main challenges for the effective use of metaproteomic data from the human microbiota (Kolmeder and De Vos, 2014). Currently, 46 bacterial GHs involved in mucin degradation have been functionally characterized, either by recombinant means, following identification of putative target genes by genomic analysis, or by purifying the bacterial enzyme following an activity screening (Table S1). Given the large diversity and complexity of mucin glycosylation, and the lack of amenable sources of mucins, functional characterization of mucin-degrading GHs often relies on the use of mucin-type oligosaccharides or synthetic substrates (such as *para*-Nitrophenol (pNP)- or 4-Methylumbelliferone (MU)-derivatized glycans) as surrogate substrates (Table S1). Owing to the paucity of purified proteins, only limited structural information is available on mucin-degrading enzymes from human gut bacteria, e.g., the absence of a GH129 crystal structure (www.cazy.org).

In addition to their catalytic domains, GHs may have one or more carbohydrate binding modules (CBMs) which mediate the adherence of CAZymes to their carbohydrate substrate. Currently, CBMs that recognize mucin glycans have been reported in families 32, 40, 47, and 51, as reviewed in Ficko-Blean and Boraston (2012a). These CBMs show specificity for terminal glycan motifs, such as Gal, GlcNAc, sialic acid, fucose, and histo-blood group antigens (Etzold and Juge, 2014). Other non-catalytic domains associated with these GHs include immunoglobulin domains, concanavalin A domains, or domains of unknown function (Table S1).

The genomic organization of mucin-degrading enzymes has been studied in few bacterial species. Bacteroidetes employ a highly organized system for glycan utilization whereby all genes involved in the degradation of dietary- or host-derived carbohydrate (GHs, sugar transport proteins, sugar sensors, regulatory proteins, etc.) are grouped together in a single PUL, as recently reviewed in Martens et al. (2009). Starch utilization systems (Sus) or Sus-like proteins have been particularly well studied in *B. thetaiotaomicron* (Martens et al., 2009). SusD proteins are cell envelope-associated proteins that mediate glycan binding, and a SusD like protein BT1043 of *B. thetaiotaomicron* has been implicated in *O*-glycan utilization of host mucins (Martens et al., 2008). Recently, the sialic acid utilizing protein NanU, a SusD family protein from *B. fragilis*, has been demonstrated to bind Neu5Ac with high affinity (Phansopa et al., 2014). Such organization in PULs is less apparent in Firmicutes. Generally the genes involved in the utilization of particular mucin glycans are clustered together in operons. For example, sialidases are often found in a cluster with other proteins involved in sialic acid catabolism (see below), and *B. longum* subsp. *infantis* has clusters dedicated to the metabolism of HMOs which share structural similarities with mucin core glycans (Sela et al., 2008).

### Sialic acid metabolism

The release of sialic acid from non-reducing ends is an initial step in the sequential degradation of mucins since the terminal location of sialic acid residues in the mucin oligosaccharide chains may prevent the action of other GHs. In bacteria, the genes involved in sialic acid metabolism are usually found clustered together forming what is denominated as a Nan cluster. Human gut bacteria that encode a Nan cluster include *R. gnavus, Anaerotruncus colihominis, Dorea formicigenerans, Dorea longicatena, F. prausnitzii, Fusobacterium nucleatum, Lactobacillus sakei, Lactobacillus plantarum*, and *Lactobacillus salivarius* (Almagro-Moreno and Boyd, 2009), *B. fragilis* (Brigham et al., 2009), and *B. breve* (Egan et al., 2014). Thus, the majority of the bacteria that harbor a Nan cluster colonize mucus regions of the human body, such as the gut, lung, bladder, or oral cavity, where sialic acid is highly abundant and can serve as a source of energy, carbon, and nitrogen (Almagro-Moreno and Boyd, 2009). However, some bacteria appear to have incomplete packages of enzymes for utilizing host sialic acids. For example, *B. thetaiotaomicron* encodes a sialidase and can release free sialic acid, but lacks the Nan operon required to consume the liberated monosaccharide and does not appear capable of consuming free sialic acid (Marcobal et al., 2011). On the other hand, *Salmonella typhimurium* and *Clostridium difficile* encode the Nan operon but each lacks the sialidase (Hoyer et al., 1992; Sebaihia et al., 2006), and thus rely on other sialidase-producing organisms to acquire this potential nutrient source from the mucosal environment (Vimr et al., 2004; Ng et al., 2013).

GH33 sialidases encoded by human gut bacteria vary in terms of their substrate specificity and enzymatic reaction. Although most of them are hydrolytic sialidases, releasing sialic acid from sialylated substrates, some display transglycosylation activities (see Table S1). For example the sialidase from *B. bifidum* JCM 1254 can transfer Neu5Ac to 1-alkanols, that of *R. gnavus* ATCC 29149 (*Rg*NanH) is an intramolecular *trans*-sialidase (IT-sialidase) which releases 2,7 anhydro-Neu5Ac specifically from α2,3-linked sialyl conjugates (Crost et al., 2013), and NanI from *Clostridium perfringens* str 13 can hydrate the inhibitor 2-deoxy-2,3-dehydro-Neu5Ac to Neu5Ac (Newstead et al., 2008). *Trans*-sialidases show specificity for α2,3 linkages, whereas hydrolytic sialidases can often cleave α2,3, 2,6, or 2,8 linkages (e.g., *B. thetaiotaomicron* sialidase BTSA). However, the substrate specificity of many sialidases remains unknown as MU-Neu5Ac is often the only tested substrate (Table S1). Furthermore, few of these purified sialidases have been enzymatically characterized using mucin as a natural substrate (Table S1). Sialidases have been identified in the genomes of infant-derived Bifidobacteria, including two intracellular sialidases from *B. longum* subsp. *infantis* ATCC 15697 (Sela et al., 2008), two predicted extracellular exo-α-sialidases of *B. bifidum* PRL 2010 (Turroni et al., 2010), and a putative sialidase from *B. breve* (Egan et al., 2014). However, the only sialidase from this group of infant-associated bacteria to be functionally characterized is SiaBb2 from *B. bifidum* JCM 1254, a strain for which the genome sequence is not yet publicly available. SiaBb2 has a strong preference for α2,6 linkages and was shown to be sufficient to confer *B. longum* 105-A with the ability to degrade HMOs (Kiyohara et al., 2011).

### Fucose metabolism

In mucins, fucosyl residues can be found at the extremity of the *O*-glycosidic chain linked to galactose by α1,2 linkages or to GlcNAc by α1,3 linkages, whereas it is most commonly linked α1,6 to the reducing, terminal β-GlcNAc in human N-linked glycans. Fucosidase-encoding genes are widely distributed in the genome of gut bacteria and generally belong to GH29 and GH95 families, which differ in their enzymatic mechanisms; GH29 enzymes retain the anomeric conformation of the glycosidic bond (Katayama et al., 2005) whereas GH95 enzymes proceed via the inverting mechanism (Nagae et al., 2007). Transcriptional data suggest that GH29 and GH95 fucosidases play a key role in the ability of *B. thetaiotaomicron* VPI-5482 (Martens et al., 2008), *B. longum* subspecies *infantis* ATCC 15697 (Sela et al., 2012), *B. bifidum* JCM 1254 (Ashida et al., 2009), and *R. gnavus* ATCC 29149 (Crost et al., 2013) to utilize mucins as a source of carbon. However, the enzymatic characterization of members of the GH95 and GH29 family is often hampered by the fact that most of these enzymes are not active against synthetic fucosyl conjugates (Katayama et al., 2005), preventing high throughput activity screening. It is notable that among 495 bacterial GH95 enzymes being listed in the CAZy database to date (02 October 2014), only two have been biochemically characterized, one from *B. longum* subspecies *infantis* ATCC 15697 and one from *B. bifidum* JCM 1254 (Table S1), both from human gut commensal strains.

The substrate specificity of GH29 and GH95 fucosidases has been predominantly characterized in *B. bifidum* JCM 1254, where GH95 AfcA was shown to be specific for α1,2 linkages (Katayama et al., 2005) and GH29 AfcB for α1,3 and α1,4 linkages (Ashida et al., 2009); together these enzymes can remove fucose at the non-reducing termini except for those that are α1,6-linked (Ashida et al., 2009). AfcA and AfcB have been shown to be sufficient to confer *B. longum* 105-A with the ability to grow on 2-fucosyllactose (2′FL), 3-fucosyllactose (3′FL) and lacto-N-fucopentaose (LNFPII) (Ashida et al., 2009). The structural basis for the specificity of AfcA has been determined (Nagae et al., 2007).

*B. thetaiotaomicron* produces multiple fucosidases that cleave fucose from host glycans, resulting in high fucose availability in the gut lumen (Xu et al., 2003). The genome of *B. thetaiotaomicron* VPI-5482 encodes five GH95 and nine GH29 genes (www.cazy.org). Two of the GH29 genes have been expressed and shown to have α-fucosidase activity and have been classified in separate sub-families, i.e., GH29-A (BT_2970) has a relaxed specificity that can accommodate pNP-fucose (pNP-Fuc), whereas GH29-B (BT_2192) is specific for branched fuco-oligosaccharides found in Lewis blood groups (also present in mucin structures, see Figure 2B) (Sakurama et al., 2012) (Table S1). The structural basis for this specificity between the two *B. thetaiotaomicron* fucosidases was first expounded by Sakurama et al. (2012) using the structures from BT_2970 (GH29-A), (Lammerts Van Bueren et al., 2010) and BT_2192 (GH29-B) (PDB 3EYP, www.rcsb.org). Further structural analysis of BT_2192 elucidated the molecular mechanisms for the binding of the branched oligosaccharides and the unusual dual specificity of this enzyme, which also acts as a β-galactosidase (Guillotin et al., 2014). More detailed analysis of the substrate specificity of fucosidases is warranted to determine why *B. thetaiotaomicron*, and indeed other resident members of the human microbiota, have evolved to produce multiple α-fucosidases.

### Blood group metabolism

Both the blood group A antigen and B antigen can be cleaved from mucin by GH98 endo-β1,4-galactosidases, these have been characterized in *Clostridium perfringens* strains 10543 and 13 (Anderson et al., 2005; Table S1; Figure 3). The only structural information about this family of enzymes comes from *Streptococcus pneumoniae* str. and reveals a (α/β)_8_ barrel (Higgins et al., 2009). Once the terminal sugars and blood group antigens are removed, the mucin core glycans are exposed to further enzymatic degradation.

### Mucin glycan core metabolism

Mucin glycan core structures are cleaved from the Ser/Thr amino acids of the mucin protein backbone by endo-α-N-acetylgalactosaminidases, with that of *B. bifidum* (EndoBF) being the founding member of GH101 (Fujita et al., 2005). These enzymes differ in their specificity toward core glycan structure types (Table S1). For example, EndoBF is specific for the core 1 glycan (Galβ1,3GalNAc) (Katayama et al., 2005) while endo-α-N-acetylgalactosaminidases from *Enterococcus faecalis* and *C. perfringens* have a broader specificity (Ashida et al., 2008; Goda et al., 2008; Koutsioulis et al., 2008). The structural basis for this specificity has been elucidated for EndoBF (Suzuki et al., 2009). The α-N-acetylgalactosaminidase from *B. bifidum* JCM 1254 is the founding member of GH129 and differs from GH101 in that it targets the Tn antigen (GalNAcα1-Ser) found in gastroduodenal mucins (Kiyohara et al., 2012). GH129 is a small family with only 58 members to date (02 Oct 14), all of which are of bacterial origin. Many of the species encoding a GH129 are associated with the infant microbiota (Kiyohara et al., 2012), although *Bacillus* sp. also contain GH129 (www.cazy.org).

GH2, GH20, and GH42 β-galactosidases have been implicated in the degradation of type-1 and type-2 HMOs. Genome analysis of several Bifidobacteria species identified common metabolic pathways for the degradation of lacto-N-biose I (Galβ1,3GlcNAc, LNB) and galacto-N-biose (Galβ1,3GalNAc, GNB), a building block of the core 1 structure of mucin-type *O*-glycan, whereas the degradation of type-2 lacto-N-neo-tetraose (Galβ1,4GlcNAcβ1,3Galβ1,4Glc, LNnT), also present in the core 2 mucin-type *O*-glycans, involves a different pathway. In Bifidobacteria, the type-1 chain (Galβ1,3GlcNAcβ1-) is likely eliminated by GH20 lacto-N-biosidases (LnbB) (Wada et al., 2008), and the released LNB incorporated into the cytosol via a GNB/LNB transporter (Suzuki et al., 2009). Despite the quite rare occurrence in nature of β-galactosidases acting on type-1 chains, close lacto-N-biosidase homologs are present in the genomes of infant-gut associated Bifidobacteria that are known to consume type 1 Lacto-N-tetraose (Galβ1,3GlcNAcβ1,3Galβ1,4Glc, LNT), these are the GH42 LNT β-galactosidases (Yoshida et al., 2012). In Bifidobacteria, the type-2 chain (Galβ1,4GlcNAcβ1-) is sequentially degraded by GH2 β-galactosidase, BbgIII, acting on LacNAc and GH20 β-N-acetylhexosaminidases, BbhI and BbhII, specific for GlcNAcβ1,3Galβ1-R (Miwa et al., 2010). Although GH2 is a very common glycosidase present in intestinal bacteria, the presence of membrane bound β-galactosidases is limited to several bifidobacterial strains. In contrast to β-galactosidases, GH20 β-N-acetylhexosaminidases are relatively rare in the genome of enteric bacteria (Miwa et al., 2010). An unusual activity was reported for a GH20 enzyme from *Prevotella* strain RS2; the enzyme cleaved terminal 6-SO_3_-GlcNAc from sulfated mucin glycans, representing a novel activity within the GH20 family (Rho et al., 2005). Other sulfatases have also been characterized (Table S1), but since these do not recognize glycosidic bonds, they are not classified in the CAZy database.

Few GH84 and GH85 β-N-acetylglucosaminidases have been characterized so far and although these enzymes have been implicated in mucin metabolism [e.g., *C. perfringens* str 13 (Ficko-Blean and Boraston, 2005), and *B. longum* NCC2705 (Schell et al., 2002)], none have been enzymatically characterized using mucin glycans as substrates (Table S1). The crystal structure of the GH84 from *B. thetaiotaomicron* VPI-5482 has been elucidated (Dennis et al., 2006), but the activity of this enzyme has been studied with protein-*O*-GlcNAc and not mucin glycans.

The GH89, α-N-acetylglucosaminidase (AgnC), from *C. perfringens* (str 13124) has been studied structurally (Ficko-Blean et al., 2008; Ficko-Blean and Boraston, 2012b) and the highly similar enzyme from *C. perfringens* str 13 has been demonstrated to be active against PGM and cell surface mucin (from adenocarcinoma AGSα4GnT cells stably expressing GlcNAcα1,4Gal as *O*-glycans on the cell surface). These studies suggest a role for AgnC in the release of terminal GlcNAc (Fujita et al., 2011) from “Class III” gastroduodenal mucins (Nakajima et al., 2003), similar to GH129 (above).

## Impact of mucin-degrading bacteria on human gut health and disease

### Spatial and temporal colonization

Members of the human gut microbiota strains typically display a subset of glycan-degrading phenotypes that equip them to target just part of the overall glycan repertoire present at certain times or locations (Koropatkin et al., 2012). The ability of mucin-degrading bacteria to forage on the diversity and abundance of glycans present in GI mucus may have a role in early colonization by providing some bacteria with an endogenous source of nutrients before the introduction of dietary glycans. Due to the chemical similarity of HMOs and *O*-linked mucin glycans, bacteria have developed common strategies for degrading these complex carbohydrates, as reported in *B. thetaiotaomicron* and Bifibobacteria (Turroni et al., 2010; Marcobal et al., 2011). Bifidobacteria possess two distinct pathways for assimilation of *O*-glycans on gastroduodenal and intestinal mucins; GH homologs involved in mucin and HMO utilization are conserved in infant-associated bifidobacteria, suggesting a significant role for their adaptation within the infant gut (Turroni et al., 2010; Kiyohara et al., 2012). A *B. thetaiotaomicron* deletion mutant for *O*-glycan utilization used in a germ-free mouse colonization experiments was outcompeted >200-fold relative to the wild-type and the complemented-mutant bacteria and pre-weaned pups selectively retained the mucin-degrading wild-type and complemented strains (Martens et al., 2008), suggesting that mucin degradation may confer an ecological advantage to the bacteria inhabiting the mammalian GI tract. *R. gnavus* is an early infant colonizer of the human intestine (Favier et al., 2002) and in the top 15 species showing abundance in both adult and infant gut-enriched genes, in line with its adaptation to the intestinal habitat throughout life (Hattori and Taylor, 2009). A recent study showed that *R. gnavus* was predominant in breast milk-/goat milk-fed microbiotas compared to a more diverse collection of Lachnospiraceae in cow milk-fed babies (Tannock et al., 2013). Taken together, these findings suggest that the ability to forage on mucin glycans in the infant GI tract may contribute to the ability of gut bacteria species to establish early colonization (Koropatkin et al., 2012).

Microbial communities that are strongly associated with the mucosa are different from those that are frequently sampled from the feces (Ouwerkerk et al., 2013), with an overrepresentation of bacteria that degrade mucins (see above). A study using germ-free mice colonized with *Escherichia coli* and the mucin degrader *B. fragilis*, revealed that only *B. fragilis* penetrates the mucus layer in the mouse colon (Huang et al., 2011). Therefore, the ability to metabolize mucin *O*-linked glycans is likely to be a key factor in determining which bacteria species adapt to the mucosal environment for *in vivo* colonization of animals. Mucosa-associated bacteria, due to their intimate proximity to the host epithelium, are likely to play a significant role in human health and disease. Indeed the intestinal epithelium directly benefits from the products of microbial metabolism by absorbing short-chain fatty acids (SCFAs) such as butyrate, propionate, and acetate which have been demonstrated to reduce the risk of GI disorders. Nutritional strategies such as the administration of prebiotics can modulate the composition of the mucosal microbes, shifting mucin degradation to distal regions, where mucin-degrading bacteria may produce metabolites influencing the host (Van Den Abbeele et al., 2011).

### Inflammation and metabolic syndromes

During the past decade there has been increasing focus on gut microbiota as an influential factor on inflammatory disease development in both humans and animals. Inflammatory bowel disease (IBD) is characterized by an increase in total mucosa-associated bacteria (Schultsz et al., 1999). IBD patients have a disproportionate representation of mucin-degrading bacteria. A ~ 100-fold and >4-fold increase in *R. torques* and *R. gnavus*, respectively, was observed in macroscopically- and histologically-normal intestinal epithelia in cases of both Crohn's disease (CD) and ulcerative colitis (UC) (Png et al., 2010). In contrast, the most abundantly detected mucolytic bacterium in controls, *A. muciniphila*, was reduced many fold in CD and in UC (Png et al., 2010). Comparison between ileal mucosa samples of healthy individuals with patients suffering from ileal CD revealed an increased abundance of *R. gnavus* with a reduced abundance of *F. prausnitzii* in CD patients (Willing et al., 2010). The same findings were observed in fecal samples from CD patients compared to unaffected controls (Sokol et al., 2009; Joossens et al., 2011). A reduction of *F. prausnitzii* in mucosa-associated microbiota of CD patients is associated with a higher risk of postoperative recurrence of ileal CD (Sokol et al., 2008). In contrast, high prevalence of aggregative, adherent *Escherichia coli* strains has been reported in the mucosa-associated microbiota of CD patients (Darfeuille-Michaud et al., 2004; Thomazini et al., 2011). In CD and UC patients only the mucosa associated population of *E. coli* is augmented and the proliferation is prominent in the ileum of CD and rectum and sigmoid of both UC and CD patients which are sites where the lesions are usually observed (De Souza et al., 2012). A recent study examining CD, UC, and irritable bowel syndrome (IBS) patients showed that mucosa-associated *F. prausnitzii* and *E. coli* co-abundance can distinguish IBS and IBD phenotypes (Lopez-Siles et al., 2014). An earlier study reported that colonic biopsies from CD-afflicted patients compared with biopsies from normal control subjects had an increase in anaerobic bacteria; in the small bowel, CD patients had an increase in the *R. gnavus* subgroup with a decrease in the *Clostridium leptum* and *Prevotella nigrescens* subgroups (Prindiville et al., 2004). A different pattern was observed in patients with active UC, where *R. gnavus* was found abundantly present in the colonic mucosa of healthy subjects but lost during active UC (Nishikawa et al., 2009). It has been hypothesized that increased mucin-degrading bacteria in IBD provide increased substrate to sustain non-mucolytic mucosa-associated bacteria, which could explain the increased total mucosa-associated bacteria in IBD. The mucin degrader *R. torques* is also frequently associated with conditions such as IBS (Malinen et al., 2010). Altogether these studies point toward an important role of mucin-degrading bacteria in modulating gut inflammatory response at the mucosal surface. It has been proposed that excessive mucin degradation by intestinal bacteria may contribute to intestinal disorders, as access of luminal antigens to the intestinal immune system is facilitated (Ganesh et al., 2013). However, it is not known whether all mucin-degraders have the same effect. For example *A. muciniphila* may possess anti-inflammatory properties, as a high proportion of the bacteria has been correlated to protection against inflammation in diseases such as type 1 diabetes mellitus (Hansen et al., 2012), IBD (Png et al., 2010), atopic dermatitis (Candela et al., 2012), autism (Wang et al., 2011), type 2 diabetes mellitus (Ellekilde et al., 2014), and obesity (Everard et al., 2013; Le Chatelier et al., 2013). *A. muciniphila* treatment can reverse fat gain, serum lipopolysaccharide (LPS) levels, gut barrier function, and insulin resistance. With regards to mucus, an increase in *A. muciniphila* has been shown to correlate with an increase in the number of goblet cells, potentially underlying the improved glucose profiles seen after *A. muciniphila* administration (Shin et al., 2014). In light of these findings, future work is warranted to gain a better understanding of the role of mucin-degraders in metabolic syndromes.

### Infection

The ability of enteric pathogens to thrive within the gut mucosal environment is intimately linked to the glycan metabolism of mucin-degrading bacteria. *S. typhimurium* accesses fucose and sialic acid within the lumen of the gut in a microbiota-dependent manner, and genetic ablation of the respective catabolic pathways reduces its competitiveness *in vivo* (Ng et al., 2013). Similarly, *C. difficile* expansion is aided by microbiota-induced elevation of sialic acid levels *in vivo*. Colonization of gnotobiotic mice with a sialidase-deficient mutant of *B. thetaiotaomicron* reduces free sialic acid levels resulting in *C. difficile* down-regulating its sialic acid catabolic pathway and exhibiting impaired expansion. These effects were reversed by exogenous dietary administration of free sialic acid (Ng et al., 2013). Furthermore, *A. muciniphila* was shown in gnotobiotic mice to exacerbate *S. typhimurium*-induced intestinal inflammation by its ability to disturb host mucus homeostasis (Ganesh et al., 2013). The GI pathogen, enterohaemorrhagic *E. coli* (EHEC), encodes a two-component system, termed FusKR, which responds to fucose and represses expression of virulence genes. During growth in mucin, *B. thetaiotaomicron* releases fucose from mucin, thereby activating the FusKR signaling cascade, modulating the virulence gene expression of EHEC (Pacheco et al., 2012). Taken together these studies indicate that mucin-derived monosaccharides made available by the microbiota profoundly influence the expansion of enteric pathogens within the gut.

## Conclusions

Bacterial-mediated mucin glycan catabolism is an important component in gut colonization which impacts on microbiota ecology and gut health. Based on the studies reported in this review, gut bacteria strains appear to rarely produce the complete set of glycosidases necessary for the degradation of mucin glycans into their constituent monosaccharides and it is thus likely that *in vivo* complete degradation of mucins in the gut relies on the cooperative action of several microbial species. In recent years an alteration of the gut microbiota structure and function has been associated with an increasing number of diseases outside and inside the gut and diet has emerged as one of the most important factors believed to affect the composition and activity of the gut microbiome. However, the systematic contribution of mucin-degraders in gut homeostasis and dysbiosis has not yet been investigated. One of the reasons is that so far only a few bacteria species have been reported and characterized as “mucin-degraders.” Expanding our knowledge into the nature of different mucin-degrading bacteria and their differential roles in the GI tract is important to help develop new therapeutic approaches aimed at restoring eubiosis in inflammatory conditions and preventing infectious diseases caused by enteric pathogens.

### Conflict of interest statement

The authors declare that the research was conducted in the absence of any commercial or financial relationships that could be construed as a potential conflict of interest.

## Abbreviations

2′FL: 2-fucosyllactose
3′FL: 3-fucosyllactose
AgnC: α-N-acetylglucosaminidase; BSM, Bovine submaxillary mucin
CAZyme: Carbohydrate-active enzyme
CBM: Carbohydrate-binding module
CD: Crohn's disease
ER: Endoplasmic reticulum
Fuc: Fucose
GAG: glycosaminoglycans
Gal: galactose
GalNAc: N-acetyl-galactosamine
GH: Glycoside hydrolase
GI: gastrointestinal
Glc: Glucose
GlcNAc: N-Acetyl-glucosamine
GNB: Galacto-N-biose
HMO: Human milk oligosaccharides
IBD: Inflammatory bowel disease
IBS: Irritable bowel syndrome
LNB: Lacto-N-biose
LnbB: Lacto-N-biosidases
LNFPII: lacto-N-fucopentaose
LNnT: Lacto-N-neo-tetraose
LNT: Lacto-N-tetraose
LPS: Lipopolysaccharide
MU: 4-Methylumbelliferone
Neu5Ac: N-Acetylneuraminic acid
PGM: Pig gastric mucin
pNP: para-Nitrophenol
pPGM: Purified pig gastric mucin
PUL: Polysaccharide-utilization loci
Pro: Proline
PTS: Proline-threonine-serine
Ser: Serine
Sus: Starch utilization system
Thr: Threonine
UC: Ulcerative colitis
VNTR: variable number of tandem repeat.
