# Supplementary material for: Mucin glycan foraging in the human gut microbiome
Source: Front Genet. 2015 Mar 19;6:81. doi: 10.3389/fgene.2015.00081 (PMC4365749; doi:10.3389/fgene.2015.00081)
Supplement: Supplementary file 1 [file Table1.DOCX]

**Table S1: Characterized mucin degrading enzymes**

| **Bacterial species and strain** | **Protein name^1^** | **Uniprot/ Genbank** | **E.C.^1^** | **PDB** | **Domains^2^** | **R/P^3^** | **Substrates tested** | **+/-^4^** | **Ref** | |
| --- | --- | --- | --- | --- | --- | --- | --- | --- | --- | --- |
| **Sialidases** | | | | | | | | | | |
| *Bacteroides fragilis* YCH46 | sialidase (BF1729) | P31206 | 3.2.1.18 |  | GH33 | R | MU-Neu5Ac | + | ([Russo et al., 1990](#_ENREF_25)) | |
| *Bacteroides thetaiotaomicron* VPI-5482 | sialidase (BtsA;BTSA;BT0455) | Q8AAK9 | 3.2.1.18 | 4BBW | GH33 | R | α2,3-, 2,6- and 2,8- linked sialylconjugates  fetuin, AGP, transferrin | +  + | ([Park et al., 2013](#_ENREF_21)) | |
| *Bifidobacterium bifidum* JCM 1254 | exo-α-sialidase (SiaBb2;BBP_0054) | BAK26854.1 | 3.2.1.18 |  | GH33 | R | MU-Neu5Ac  α2,3-, 2,6- and 2,8- linked sialylconjugates (2,3- linkages preferred),  gangliosides, fetuin, PGM, hen egg yolk N-glycans  also transfers Neu5Ac to 1-alkanols | +  +  +  + | ([Kiyohara et al., 2011](#_ENREF_16)) | |
| *Clostridium perfringens* A99 | sialidase 1 'small' | P10481 | 3.2.1.18 |  | GH33 | R | MU-Neu5Ac | + | ([Roggentin et al., 1995](#_ENREF_24)) | |
| *Clostridium perfringens* ATCC 10543 | sialidase 2 (NanH) | Q59311 | 3.2.1.18 |  | GH33 | R | MU-Neu5Ac | + | ([Chien et al., 1996](#_ENREF_3)) | |
| *Clostridium perfringens* ATCC 13124^5^ | sialidase (CPF_0721) | Q0TT67 | 3.2.1.18 | 4L2E | CBM40, GH33 | R | MU-Neu5Ac | + | ([Lee et al., 2014](#_ENREF_18)) | |
| *Clostridium perfringens* str 13^5^ | exo-α-sialidase (NanI;CPSA;CPE0725) | Q8XMG4 | 3.2.1.18 | 2BF6 2VK5 2VK6 2VK7 | CBM40, GH33 | R | Fetuin, BSM, colominic acid, bovine brain gangliosides  Can also hydrate 2-deoxy-2,3-dehydro-Neu5Ac acid to Neu5Ac | +  + | ([Roggentin et al., 1995](#_ENREF_24);[Newstead et al., 2008](#_ENREF_20)) | |
| *Clostridium tertium* | sialidase (NanH;SiaH) | P77848 | 3.2.1.18 |  | CBM40, GH33 | R | MU-Neu5Ac | + | ([Grobe et al., 1998](#_ENREF_11)) | |
| *Salmonella typhimurium* TA262 | sialidase (NanH;STSA) | P29768 |  | 1DIL 1DIM 2SIL 2SIM 3SIL | GH33 | R | MU-Neu5Ac | + | ([Taylor et al., 1992](#_ENREF_29)) | |
| *Vibrio cholerae* 569B 395 | sialidase / neuraminidase (NanH;VcNA;VCSA) | P37060/P0C6E9 | 3.2.1.18 | 1KIT 1W0O 1W0P | CBM40, GH33 | R | MU-Neu5Ac, glycans of C57BL/6 mouse fibroblasts | + | ([Crennell et al., 1994](#_ENREF_4)) | |
| *Ruminococcus gnavus* ATCC 29149 | *Rg*NanH^2^ | A7B557 | 4.2.1.15 |  | CBM40, GH33 | R | MU-Neu5Ac, α2,3-, AGP, Fetuin  α2,6- linkages, asialofetuin  Releases 2,7 anhydro-Neu5Ac | +  -  + | Tailford, personal communication | |
| *Akkermansia muciniphila* ATCC BAA-835 | Amuc_0625^2^ | B2UPI5 | 3.2.1.18 |  | GH33 | R | MU-Neu5Ac, α2,3-, AGP, Fetuin  α2,6- linkages, asialofetuin | +  - | Tailford, personal communication | |
|  | Amuc_1835^2^ | B2UN42 | 3.2.1.18 |  | GH33 | R | MU-Neu5Ac, α2,3-, AGP, Fetuin  α2,6- linkages, asialofetuin | +  - | Tailford, personal communication | |
| **Fucosidases** | | | | | | | | | | |
| *Bacteroides thetaiotaomicron* VPI-5482 | α-1,3/1,4-L-fucosidase / α-galactosidase (BT2192;BT_2192) | Q8A5P6 | 3.2.1.111 | 3EYP 4OUE 4OZO | GH29-B^7^ | R | Le^a^, Le^b^, Le^x^, Le^y^, pNP-Gal  pNP-Fuc,unbranched fuco-oligosaccharides, other pNP-monosaccharides | +  - | ([Sakurama et al., 2012](#_ENREF_26);[Guillotin et al., 2014](#_ENREF_12)) | |
|  | α-L-fucosidase (Fuc2970;BtFuc2970;BT2970;BT_2970) | Q8A3I4 | 3.2.1.51 | 2WVS  2WVT 2WVU  2WVV  2XIB 2XII 4J27  4J28 4JFS 4JFT 4JFU 4JFV 4JFW | GH29-A^7^  CBM32 | R | pNP-Fuc, Fucα1-3GlcNAc, Fucα1-4GlcNAc, but not α1,2- or α1,6-, nor β linkages | +  - | ([Sakurama et al., 2012](#_ENREF_26)) | |
| *Bifidobacterium bifidum* JCM 1254 | α-1,3/4-L-fucosidase (AfcB;BbAfcB) | BAH80310.1 | 3.2.1.111 |  | GH29, CBM32, FIVAR | R | 3-FL, Le^a^, Le^b^, Le^X^, Le^y^, LNFPII, LNFPIII  2’-FL, Fucα1-6GlcNAc, sialyl Le^X^, pNP-Fuc | +  - | ([Ashida et al., 2009](#_ENREF_2)) | |
| *Bifidobacterium longum* subsp. *infantis* ATCC 15697 | α-1,3/1,4-L-fucosidase (Blon_2336) | B7GNN8 | 3.2.1.51 3.2.1.111 | 3MO4 3UES  3UET | GH29, CBM32 |  | CMP-Fuc, 3’FL,  2’FL or Fucα1-2Gal  LNFPI and LNFPIII | +  -  + | ([Sela et al., 2012](#_ENREF_27)) | |
|  | α-L-fucosidase (Blon_0248) | B7GTT5 | 3.2.1.51 |  | GH29 |  | CMP-Fuc, LNFPIII  3’FL, 2’FL, Fucα1-2Gal, LNFPI | +  - | ([Sela et al., 2012](#_ENREF_27)) | |
|  | α-L-fucosidase (Blon_0426) | B7GN40 | 3.2.1.51 |  | GH29 |  | CMP-Fuc, LNFPIII  3’FL, 2’FL or Fucα1-2Gal, LNFPIII | +  - | ([Sela et al., 2012](#_ENREF_27)) | |
|  | Blon_0346 | ACJ51470.1 |  |  | GHnc | R | CMP-Fuc, Fucα1-2Gal  3’FL, 2’FL, LNFP I, LNFP III | +  - | ([Sela et al., 2012](#_ENREF_27)) | |
|  | α-1,2-L-fucosidase (Blon_2335) | B7GNN7 | 3.2.1.51 |  | GH95 | R | CMP-Fuc, 2’FL, 3’FL, Fucα1-2Gal  LFNPI, LFNPIII | +  + | ([Sela et al., 2012](#_ENREF_27)) | |
| *Bifidobacterium bifidum* JCM 1254 | α-1,2-L-fucosidase (AfcA;AfuA;BbAfcA) | AAQ72464.1 | 3.2.1.63 | 2EAB 2EAC  2EAD 2EAE | GH95,  Ig | R | terminal α1,2- fucosidic linkages of various oligosaccharides,  α1,3- or α1,6- linkages, nor synthetic substrates.  sugar chains of glycoproteins, including PGM | +  -  + | ([Katayama et al., 2005](#_ENREF_14)) | |
| **Blood-group endo-β-1,4-galactosidases (GH98)** | | | | | | | | | | |
| *Clostridium perfringens ATCC 10543* | blood group A- and B-cleaving endo-β-galactosidase (Endo-ABase;EabC) | Q6RUF5 | 3.2.1.102 |  | CBM51, GH98 | P &R | Blood group A and B trisaccharides  PGM, human ovarian cyst glycoprotein | +  + | Anderson, 2002 | |
| *Clostridium perfringens str. 13* | blood group A- and B-cleaving endo-β-galactosidase (Cpe0329) | BAB80035.1 | 3.2.1.102 |  | CBM51 | - | Presumed to be as Q6RUF5 as identical sequence |  | Anderson, 2002 | |
| **Mucin core GHs (GH101, GH129, unclassified)** | | | | | | | | | | |
| *Bifidobacterium longum subsp. longum* JCM 1217 | core 1 endo-α-N-acetylgalactosaminidase (EngBF;BLLJ_0168) | Q3T552 | 3.2.1.97 | 2ZXQ | GH101 | R | Galβ1-3GalNAcα1-pNP, asialofetuin  other pNP substrates, fetuin | +  - | ([Fujita et al., 2005](#_ENREF_7);[Katayama et al., 2005](#_ENREF_14)) | |
| *Clostridium perfringens* ATCC 13124 | core 1 endo-α-N-acetylgalactosaminidase (EngCP;CPF_0685) | Q0TTA3 | 3.2.1.97 |  | GH101 | R | Core 1-, Core 2-, Core 8-, GalNAc- conjugates | + | ([Koutsioulis et al., 2008](#_ENREF_17)) | |
| *Clostridium perfringens* str 13 | endo-α-N-acetylgalactosaminidase (EngCP;CPE0693 | Q8XMJ5 | 3.2.1.97 |  | GH101 | R | Core 1-, Core 2-, Core 8-, GalNAc- conjugates  asialomucin and asialofetuin  sialylated mucin or fetuin | +  +  - | ([Ashida et al., 2008](#_ENREF_1)) | |
| *Enterococcus faecalis* NBRC3971 | core 1 / core 3 endo-α-N-acetylgalactosaminidase (EngEF;EndoEF) (partial) | B5UB72 | 3.2.1.97 |  | GH101 | R | Core 1, Core 2, Gal-Core 2, Core 3  core 6, fetuin  mucin glycopeptides, asialomucin, asialofetuin  transfer of core 1-disaccharides and core 2-trisaccharides to alkanols generating alkyl-oligosaccharides. | +  -  +  + | ([Goda et al., 2008](#_ENREF_9)) | |
| *Ruminococcus torques* IX-70 | α-N-acetylgalactosaminidase | No sequence info | 3.2.1.49 |  | ^2^ | P | blood group A and AB mucin glycoproteins | + | ([Hoskins et al., 1997](#_ENREF_13)) | |
| *Bifidobacterium bifidum* JCM1254 | α-N-acetylgalactosaminidase NagBb | BAL14929.1 | 3.2.1.49 |  | GH129 | R | GalNAcα1-Ser (Tn antigen)  sialylated Tn antigen.  GalNAcα1-3Galβ1-4Glc  GalNAcα1-UDP, GalNAcα1-pNP,  other monosaccharide-pNP substrates.  GalNAcα1-DMT, pNP-; Core 1, Core 3, Core 7 and Core 8,  pNP-; A trisaccharide, Core 2, Core 4, Core 5 or Core 6  Transglycosylation activity of GalNac from GalNAcα1-pNP and GalNAcα1-DMT | +  -  +  +  -  +  -  + |  | |
| **Mucin core GHs and sulfatases (GH20, GH2, GH42, unclassified)** | | | | | | | | | | |
| *Bifidobacterium bifidum* JCM 1254 | lacto-N-biosidase (LnbB) | B3TLD6 | 3.2.1.140 | 4H04 4JAW | GH20 | R | Galβ1-3GlcNAcβ-pNP, LNT,  Galβ1-3GalNAcβ-pNP  GlcNAcβ-pNP, GalNAcβ-pNP, α-linked pNP glycoconjugates, other HMOs | +  +  - | ([Wada et al., 2008](#_ENREF_30)) | |
|  | lacto-N-triose II β-N-acetylhexosaminidase (BbhI) | BAI94822.1 | 3.2.1.52 |  | CBM32, CBM32, GH20, CBM32, Ig, Ig | R | GlcNAcβ1-pNP, LNTII, pNP-α-Core 2,  GalNAcβ1-pNP (low), GlcNAcβ1-2Man  GlcNAcβ1-3Man, GlcNAcβ1-4Glc, GlcNAcβ1-4MurNAc | +  +/-  - | ([Miwa et al., 2010](#_ENREF_19)) | |
|  | β-N-acetylhexosaminidase (BbhII) | BAI94823.1 | 3.2.1.52 |  | CBM32, GH20 | R | GlcNAcβ1-pNP, GalNAcβ1-pNP, pNP-α-Core 2  LNTII, GlcNAcβ1-2Man, GlcNAcβ1-3Man, GlcNAcβ1-4Glc, GlcNAcβ1-4MurNAc | +  - | ([Miwa et al., 2010](#_ENREF_19)) | |
|  | lacto-N-neotetraose β-galactosidase (BbgIII) | BAI94821.1 | 3.2.1.23 |  | GH2, Ig, GH2, Ig, Ig, CBM32, CBM32, 3 x FIVAR | R | pNP-β-Gal, Lac, LacNAc, LNnT, LNH, allo-LacNAc, Lactulose  LNB, LNT, 3-FL, Le^X^, LNFP III | +  - | ([Miwa et al., 2010](#_ENREF_19)) | |
| *Bifidobacterium longum* subsp. *infantis* ATCC 15697 | β-galactosidase 2A (Blon_2334) (Bga2A) | B7GNN6 | 3.2.1.23 |  | GH2 | R | pNP-β-Gal, pNP-β-Fuc, Lac, LacNAc, LNnT,  LNB, LNT  other pNP glycoconjugates | +  +  +/-  - | ([Yoshida et al., 2012](#_ENREF_32)) | |
|  | β-galactosidase 42A (Blon_2016) (Bga42A) | B7GUD7 | 3.2.1.23 |  | GH42 | R | pNP-β-Gal, pNP-β-Fuc, Lac, LacNAc, LNnT, LNB, LNT,  other pNP glycoconjugates | +  +  - | ([Yoshida et al., 2012](#_ENREF_32)) | |
|  | β-galactosidase 42B (Blon_2123) (Bga42B) | B7GUN8 | 3.2.1.23 |  | GH42 | R | pNP-β-Gal, pNP-β-Fuc, Lac,  LacNAc, LNB, LNT  LNnT, other pNP substrates | +  +/-  - | ([Yoshida et al., 2012](#_ENREF_32)) | |
|  | β-galactosidase 42C (Blon_2416) (Bga42C) | B7GNW4 | 3.2.1.23 |  | GH42 | R | pNP-β-Gal, pNP-β-Fuc, Lac, LacNAc, LNnT,  LNB, LNT, other pNP substrates | +  +  - | ([Yoshida et al., 2012](#_ENREF_32)) | |
| *Bifidobacterium bifidum* NCIMB 41171 | β-galactosidase P2 (BbgII) | A5A2I5 | 3.2.1.23 |  | GH42 | R | Lactose, Galβ1-6Gal, LacNAc, Lactulose, Galβ1-4Gal, Galβ1-4Galβ1-4Glc,  LNB, or 3FL | +  - | ([Goulas et al., 2009](#_ENREF_10)) | |
|  | β-galactosidase P1 (BbgI;Bbg1) | Q0ZI53 | 3.2.1.23 |  | GH2 | R | Lactose, Galβ1-6Gal, LacNAc, Lactulose,  Galβ1-4Gal, Galβ1-4Galβ1-4Glc, LNB, or 3FL | +  - | ([Goulas et al., 2009](#_ENREF_10)) | |
|  | β-galactosidase S1 (BbgIII) | A4K5H9 | 3.2.1.23 |  | GH2 | R | Lactose, Galβ1-6Gal, LacNAc, Lactulose, Galβ1-4Gal, Galβ1-4Galβ1-4Glc.  LNB, or 3FL | +  - | ([Goulas et al., 2009](#_ENREF_10)) | |
|  | β-galactosidase K2 (BbgIV) | Q0ZII7 | 3.2.1.23 |  | GH2 | R | Lactose, Galβ1-6Gal, LacNAc, Lactulose,  Galβ1-4Gal, Galβ1-4Galβ1-4Glc, LNB, or 3FL | +  - | ([Goulas et al., 2009](#_ENREF_10)) | |
| *Helicobacter pylori* (Strain NS) | glycosulfatase | No sequence info | NS |  | ^6^ | P | GlcNAc-6-sulfate, Gal-6-sulfate, Glc-6-sulfate  Gal-3-sulfate | +  - | ([Slomiany et al., 1992](#_ENREF_28)) | |
| *Prevotella* sp RS2 | **Mucin-desulfating sulfatase MdsA** | **Q9L5W0** | 3.2.6.14 |  | ^6^ | P | GlcNac-6-sulfate, Gal-6-sulfate, Gal-3-sulfate | + | ([Wright et al., 2000](#_ENREF_31)) | |
|  | mucin-desulfating glycosidase / sulfoglycosidase / β-6-SO3-N-acetylglucosaminidase (Sgl) | Q5MAH5 | 3.2.1.- |  | GH20 | R | Sulfomucin, pNP-2-acetamido-2-deoxy-beta-D-glucopyranoside 6-sodium sulfate | + | ([Rho et al., 2005](#_ENREF_23)) | |
| **Mucin core GHs (GH84, GH85 and GH89)** | | | | | | | | | | |
| *Bacteroides thetaiotaomicron* VPI-5482 | β-N-acetylglucosaminidase (BtOGA;BtGH84;BT4395;BT_4395) | Q89ZI2 | 3.2.1.169 | 2CHN  2CHO 2J47 2J4G 2JIW 2VVN 2VVS 2W4X 2W66 2W67 2WCA 2WZH 2WZI 2X0H 2XJ7 2XM1 2XM2 4AIS 4AIU | GH84 | R | pNP-O-GlcNAc  glycoproteins with an O-GlcNAc linkage | +  + | | ([Dennis et al., 2006](#_ENREF_5)) |
| *Clostridium perfringens* ATCC 13124 | hyaluronidase (NagJ;CpNagJ;CPF_1442) | Q0TR53 | 3.2.1.52 | 2J62 2V5C 2V5D 2VUR 2WB5 2X0Y 2YDQ 2YDR 2YDS | GH84, CBM32 | R | pNP-O-GlcNAc  glycoproteins with an O-GlcNAc linkage | +  + | | ([Rao et al., 2006](#_ENREF_22)) |
|  | α-N-acetylglucosaminidase (AgnC;CpGH89;CPF_0859) | Q0TST1 | 3.2.1.50 | 2VC9 2VCA 2VCB 2VCC 4A4A | CBM32-1,GH89,- CBM32-2-5, FIVAR x4, UNK, Fn3 | R | Presumed to be the same as Q8XM24 |  | | ([Ficko-Blean et al., 2008](#_ENREF_6)) |
| *Clostridium perfringens*  str 13 | α-N-acetylglucosaminidase AgnC;CPE0866 | Q8XM24 | 3.2.1.50 |  | CBM32-1,GH89,- CBM32-2-5, FIVAR x4, UNK, Fn3 | R | GlcNAcα1,4Galβ1pMP and  galactose, PGM, cell surface mucin  GlcNAcα1-2Galβ1pMP, GlcNAcα1-3Galβ1pMP, GlcNAcα1-6Galβ1pMP, GlcNAcα1-4GlcAβ1pMP | +  - | | ([Fujita et al., 2011](#_ENREF_8)) |
| *Bifidobacterium bifidum* JCM 1254 | α-N-acetylgalactosaminidase NagBb | BAL14929.1 | 3.2.1.49 |  | GH129 | R | GalNAcα1-Ser (Tn antigen)  sialylated Tn antigen, monosaccharide-pNP substrates  GalNAcα1-3-Galβ1-4-Glc  GalNAcα1-UDP, GalNAcα1-pNP  GalNAcα1-DMT, pNP-; Core 1, Core 3, Core 7 and Core 8  pNP-; A trisaccharide, Core 2, Core 4, Core 5 or Core 6  Transglycosylation activity of GalNAc from GalNAcα1-pNP and GalNAcα1-DMT | +  -  +  +  +  -  + | | ([Kiyohara et al., 2012](#_ENREF_15)) |

**Footnotes**

^1^The protein names and E.C. numbers are given as is listed in CAZy. ^2^The domains are mostly taken from the CAZy database although additional domains are added if they are described in the literature and are thought to be of relevance to this review-for further domain information please see Pfam or similar databases. ^3^R/P refers to whether the characterisation is carried out with recombinant (R) or purified (P) enzymes. ^4^This column indicates whether the enzyme is active (+) or not (-) against the substrates tested, where activity is very low the symbol “+/-“ is used. ^5^NB these strains are “flesh-eating” strains isolated from gangrene rather than gut bacteria but are included because more biochemical data is available. ^6^Not in CAZy.^7^See text for –A and –B classification.

**Abbreviations**

2’-FL-2’ fucosyllactose, 3-FL-3 fucosyllactose, AGP-α1-Acid glycoprotein, A-trisaccharide-Blood group A trisaccharide-GalNAcα1-3-(Fucα1-2)Gal, BSM-bovine submaxillary mucin, CMP-2-chloro-4-nitrophenyl-α-[SCAP], Core 1-8 see Fig 2, DMT-4,6-dimethoxy-1,3,5-triazin-2-yl, FIVAR-found in various architectural regions, FN3-module sharing distant identity with fibronectin type III domains, Fuc-Fucose, Gal -Galactose, GalNAc-N-acetylgalactosamine, GHnc-Glycoside Hydrolase-not classified, Glc-Glucose, GlcNAc-N-acetylglucosamine, HMO-human milk oligosaccharide, Ig-immunoglobulin domain, Lac-Lactose- Galβ1-4Glc, LacNAc-N-acetyllactosamine- Galβ1-4GlcNAc, Le^a^ -Lewis a- Galβ1-4(Fucα1-3)Glc (3-FL), Galβ1-3(α1-4)GlcNac, Le^b^ -Lewis b- Fucα1-2Galβ1-3(Fucα1-4)GlcNAc, Le^x^ -Lewis x- Galβ1-4(Fucα1-3)GlcNAc , Le^y^-Lewis y-Fucα1-2Galβ1-4(Fucα1-3)GlcNAc, LNB-Galβ1-3GlcNAc, LNFP-lacto-*N*-fucopentaose Type I-α1-3, II-α1-4, III-α1-2, LNnT-Lacto-N-neotetraose-Galβ1-4GlcNacβ1-3Galβ1-4Glc, LNT-Lacto-N-tetraose, Galβ1,3,GlcNAcβ1,3,Galβ1,4Glc, LNTII-GlcNAcβ1-3Galβ1-4Glc, Man-Mannose, MU-methylumbelliferyl, MurNAc-N-acetylmuramic acid, Neu5Ac-N-acetyl neuraminic acid, NS-not specified, PGM-porcine gastric mucin, pNP-*para*-Nitrophenol, UNK -module having unknown function and little sequence identity with other proteins of known function, X82-module of unknown function which group together in a family known as X82.

**E.C. numbers**

E.C. 3.1.6.15- **N-acetylglucosamine-6-sulfatase,** E.C. 3.2.1.18-sialidase, E.C.3.2.1.23-β-galactosidase, E.C.3.2.1.49-α-N-acetylgalactosaminidase, **E.C. 3.2.1.49-α-N-acetylgalactosaminidase,** E.C.3.2.1.50-α-N-acetylglucosaminidase, E.C.3.2.1.51-α-L-fucosidase, E.C.3.2.1.52-β-N-acetylhexosaminidase, E.C.3.2.1.52-β-*N*-acetylhexosaminidase, E.C. 3.2.1.63-**1,2-α-L-fucosidase,** E.C. 3.2.1.97-**Endo-α-N-acetylgalactosaminidase, E.C. 3.2.1.102-**blood-group-substance endo-1,4-β-galactosidase, E.C.3.2.1.111-1,3-α-L-fucosidase, E.C. 3.2.1.140-lacto-N-biosidase, E.C. 3.2.1.169 protein *O*-GlcNAcase, E.C. 4.2.2.15-anhydrosialidase.

**Supplementary references**

Chien, C.H., Shann, Y.J., and Sheu, S.Y. (1996). Site-directed mutations of the catalytic and conserved amino acids of the neuraminidase gene, nanH, of Clostridium perfringens ATCC 10543. *Enzyme Microb Technol* 19**,** 267-276.

Crennell, S., Garman, E., Laver, G., Vimr, E., and Taylor, G. (1994). Crystal structure of Vibrio cholerae neuraminidase reveals dual lectin-like domains in addition to the catalytic domain. *Structure* 2**,** 535-544.

Goulas, T., Goulas, A., Tzortzis, G., and Gibson, G.R. (2009). Comparative analysis of four beta-galactosidases from Bifidobacterium bifidum NCIMB41171: purification and biochemical characterisation. *Appl Microbiol Biotechnol* 82**,** 1079-1088. doi: 10.1007/s00253-008-1795-5.

Grobe, K., Sartori, B., Traving, C., Schauer, R., and Roggentin, P. (1998). Enzymatic and molecular properties of the Clostridium tertium sialidase. *J Biochem* 124**,** 1101-1110.

Hoskins, L.C., Boulding, E.T., and Larson, G. (1997). Purification and characterization of blood group A-degrading isoforms of alpha-N-acetylgalactosaminidase from Ruminococcus torques strain IX-70. *J Biol Chem* 272**,** 7932-7939.

Lee, Y., Ryu, Y.B., Youn, H.S., Cho, J.K., Kim, Y.M., Park, J.Y., Lee, W.S., Park, K.H., and Eom, S.H. (2014). Structural basis of sialidase in complex with geranylated flavonoids as potent natural inhibitors. *Acta Crystallogr D Biol Crystallogr* 70**,** 1357-1365. doi: 10.1107/S1399004714002971.

Park, K.H., Kim, M.G., Ahn, H.J., Lee, D.H., Kim, J.H., Kim, Y.W., and Woo, E.J. (2013). Structural and biochemical characterization of the broad substrate specificity of Bacteroides thetaiotaomicron commensal sialidase. *Biochim Biophys Acta* 1834**,** 1510-1519. doi: 10.1016/j.bbapap.2013.04.028.

Rao, F.V., Dorfmueller, H.C., Villa, F., Allwood, M., Eggleston, I.M., and Van Aalten, D.M. (2006). Structural insights into the mechanism and inhibition of eukaryotic O-GlcNAc hydrolysis. *Embo j* 25**,** 1569-1578. doi: 10.1038/sj.emboj.7601026.

Roggentin, P., Kleineidam, R.G., and Schauer, R. (1995). Diversity in the properties of two sialidase isoenzymes produced by Clostridium perfringens spp. *Biol Chem Hoppe Seyler* 376**,** 569-575.

Russo, T.A., Thompson, J.S., Godoy, V.G., and Malamy, M.H. (1990). Cloning and expression of the Bacteroides fragilis TAL2480 neuraminidase gene, nanH, in Escherichia coli. *J Bacteriol* 172**,** 2594-2600.

Taylor, G., Vimr, E., Garman, E., and Laver, G. (1992). Purification, crystallization and preliminary crystallographic study of neuraminidase from Vibrio cholerae and Salmonella typhimurium LT2. *J Mol Biol* 226**,** 1287-1290.

Wright, D.P., Rosendale, D.I., and Robertson, A.M. (2000). Prevotella enzymes involved in mucin oligosaccharide degradation and evidence for a small operon of genes expressed during growth on mucin. *FEMS Microbiol Lett* 190**,** 73-79.
